# Supplementary material for: Genetic validation of bipolar disorder identified by automated phenotyping using electronic health records
Source: Transl Psychiatry. 2018 Apr 18;8:86. doi: 10.1038/s41398-018-0133-7 (PMC5904248; doi:10.1038/s41398-018-0133-7)
Supplement: Supplementary file 1 — Supplementary Information [file 41398_2018_133_MOESM1_ESM.docx]

**Supplementary Information**

**Genetic validation of bipolar disorder identified by automated phenotyping using electronic health records**

Chia-Yen Chen^1-5^, Phil H. Lee^1,3-5^, Victor M. Castro^1,6,7^, Jessica Minnier^8^, Alexander W. Charney^9-11^, Eli A. Stahl^9,10^, Douglas M. Ruderfer^12^, Shawn N. Murphy^7,13,14^, Vivian Gainer^7^, Tianxi Cai^14,15^, Ian Jones^16^, Carlos Pato^17^, Michele Pato^17^, Mikael Landén^18,19^, Pamela Sklar^9-11^, Roy H. Perlis^1,3-6^, Jordan W. Smoller^1,3-5^

1. Psychiatric and Neurodevelopmental Genetics Unit, Massachusetts General Hospital, 185 Cambridge St., Boston, MA 02114, USA
2. Analytic and Translational Genetics Unit, Center for Human Genetic Research, Massachusetts General Hospital, 185 Cambridge St., Boston, MA 02114, USA
3. Center for Genomic Medicine, Massachusetts General Hospital, 185 Cambridge St, Boston, MA 02114, USA.
4. Department of Psychiatry, Massachusetts General Hospital, 55 Fruit Street, Boston, MA 02114, USA
5. Broad Institute of MIT and Harvard, 75 Ames Street, Cambridge, MA 02142, USA.
6. Center for Experimental Drugs and Diagnostics, Massachusetts General Hospital, 55 Fruit Street, Boston, MA 02114, USA
7. Partners Research Information Systems and Computing, Partners HealthCare System, One Constitution Center, Charlestown, MA 02129, USA
8. Oregon Health & Sciences University, 3181 SW Sam Jackson Park Rd, Portland, OR 97239, USA
9. Department of Psychiatry, Icahn School of Medicine at Mount Sinai, One Gustave L. Levy Place, New York, NY 10029, USA
10. Institute for Genomics and Multiscale Biology, Department of Genetics and Genomic Sciences, Icahn School of Medicine at Mount Sinai, One Gustave L. Levy Place, New York, NY 10029, USA
11. Friedman Brain Institute, Department of Neuroscience, Icahn School of Medicine at Mount Sinai, One Gustave L. Levy Place, New York, NY 10029, USA
12. Division of Genetic Medicine, Vanderbilt University Medical Center, Nashville, TN 37212, USA
13. Department of Neurology, Massachusetts General Hospital, 55 Fruit Street, Boston, MA 02114, USA
14. Department of Biomedical Informatics, Harvard Medical School, 10 Shattuck Street, Boston, MA 02115, USA
15. Department of Biostatistics, Harvard T.H. Chan School of Public Health, 677 Huntington Ave, Boston, MA 02115, USA
16. National Centre for Mental Health, MRC Centre for Neuropsychiatric Genetics and Genomics, Cardiff University, Cardiff, CF24 4HQ, UK
17. SUNY Downstate Medical Center, Brooklyn, NY 11203, USA
18. Institute of Neuroscience and Physiology, Department of Psychiatry and Neurochemistry, The Sahlgrenska Academy, University of Gothenburg, Gothenburg, Sweden
19. Department of Medical Epidemiology and Biostatistics, Karolinska Institutet, Stockholm, Sweden

Correspondence to:

Jordan W. Smoller, MD, ScD

Simches Research Building

185 Cambridge St.

Boston, MA 02114

Phone: 617-724-0835; Fax: 617-643-3080

Email: [jsmoller@mgh.harvard.edu](mailto:jsmoller@hms.harvard.edu)

**Supplementary Figure 1.** Venn diagram showing bipolar case frequency identified by each EHR-based algorithm


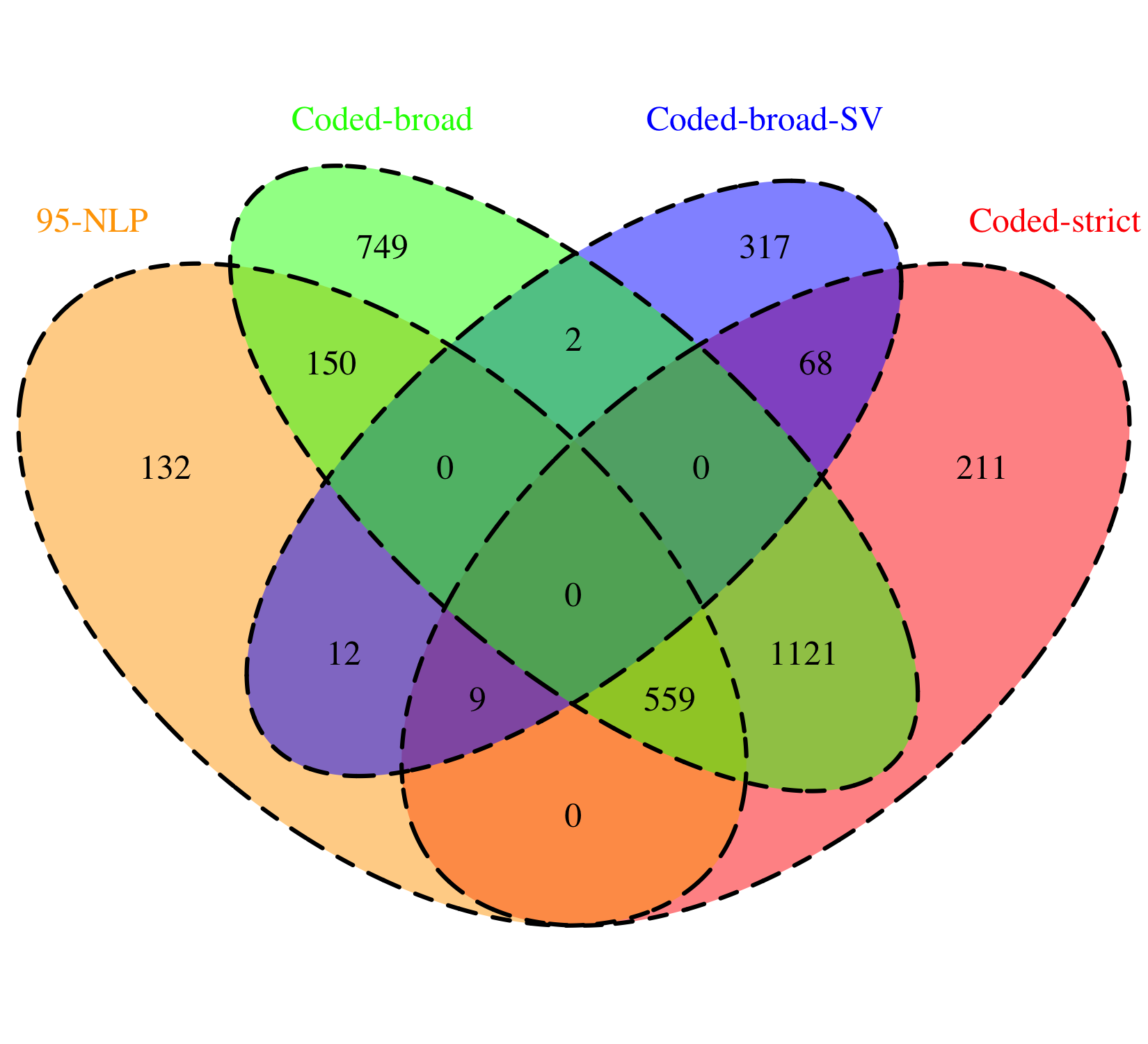


**Supplementary Figure 2.** Distribution of distance to average European HapMap3 reference samples based on PC1 and PC2. We used distance < 0.01 as a cut-off to selected European population study samples.


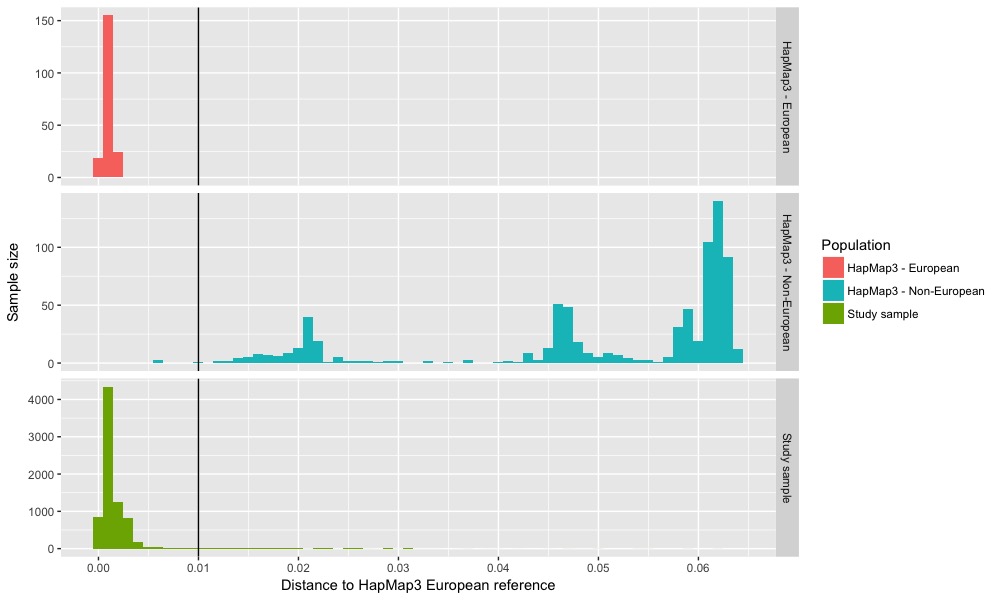


**Supplementary Figure 3.** PCA plot of study samples combined with HapMap3 reference samples. European population study samples were selected based on the distance to average European HapMap3 reference samples (Supplementary figure 1).


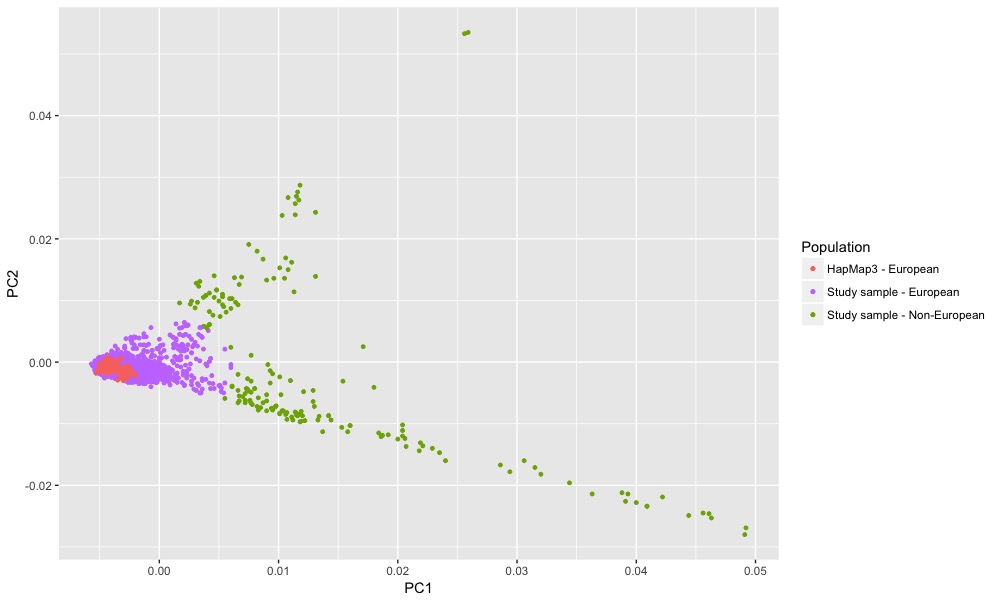


**Supplementary Figure 4.** PCA plot of European population study samples. The PCA plot showed typical population structure of European population samples in the U.S., which includes a Northwest-Southeast European cline and a separate Ashkenazi Jewish cluster. The color-coding showed the bipolar disorder cases and controls by genotyping batches.


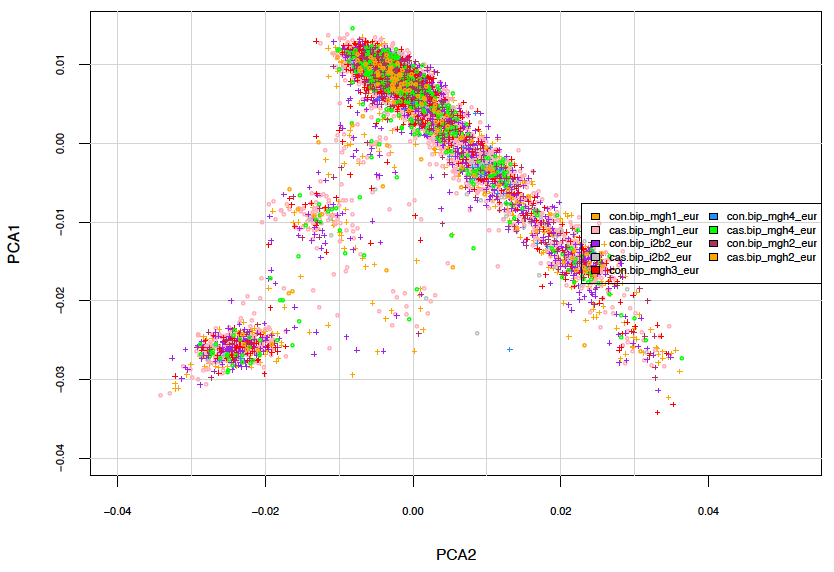


**Supplementary Figure 5.** Percentage of bipolar disorder subtypes for different case ascertainment methods. SAB: schizoaffective disorder bipolar type. NOS: not otherwise specified.


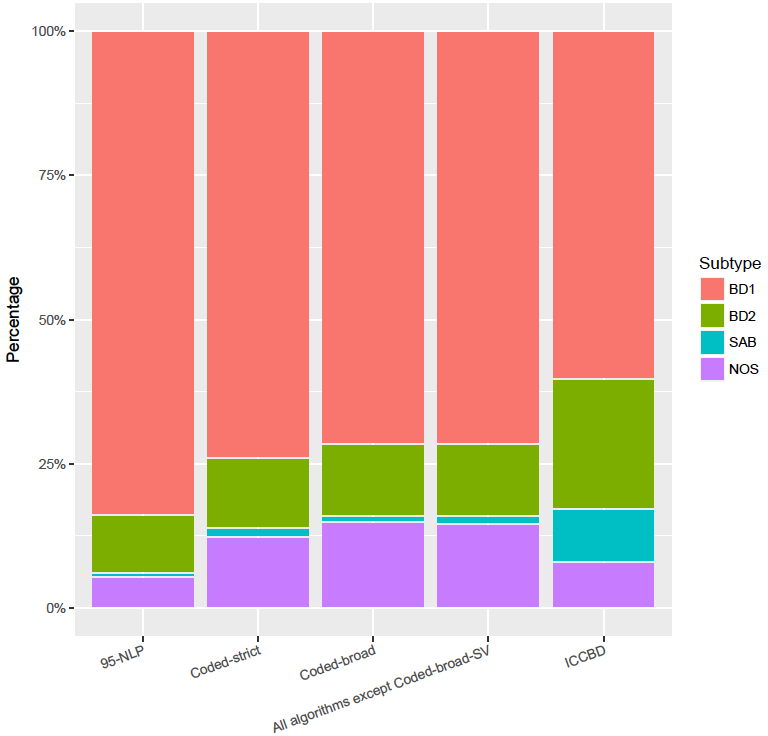


**Supplementary Table 1.** SNP-based cross-phenotype genetic correlation (r_g_) between bipolar disorder and schizophrenia (SCZ), major depressive disorder (MDD), subjective well-being, and mean platelet volume (MPV). SE: standard error.

| Phenotype 1 | Phenotype 2 | r_g_ | SE | P-value |
| --- | --- | --- | --- | --- |
| 95-NLP | Schizophrenia | 0.51 | 0.110 | 4.10E-06 |
| 95-NLP | Major depressive disorder | 0.53 | 0.199 | 7.10E-03 |
| 95-NLP | Subjective well-being | -0.25 | 0.136 | 6.40E-02 |
| 95-NLP | Mean platelet volume | 0.01 | 0.124 | 9.38E-01 |
| Coded-strict | Schizophrenia | 0.61 | 0.158 | 1.00E-04 |
| Coded-strict | Major depressive disorder | 0.82 | 0.225 | 3.00E-04 |
| Coded-strict | Subjective well-being | -0.32 | 0.151 | 3.22E-02 |
| Coded-strict | Mean platelet volume | 0.12 | 0.159 | 4.61E-01 |
| Coded-broad | Schizophrenia | 0.49 | 0.099 | 7.99E-07 |
| Coded-broad | Major depressive disorder | 0.67 | 0.175 | 1.00E-04 |
| Coded-broad | Subjective well-being | -0.30 | 0.127 | 1.98E-02 |
| Coded-broad | Mean platelet volume | 0.07 | 0.139 | 5.90E-01 |
| All algorithms except coded-broad-SV | Schizophrenia | 0.55 | 0.107 | 2.36E-07 |
| All algorithms except coded-broad-SV | Major depressive disorder | 0.77 | 0.186 | 3.97E-05 |
| All algorithms except coded-broad-SV | Subjective well-being | -0.27 | 0.127 | 3.19E-02 |
| All algorithms except coded-broad-SV | Mean platelet volume | 0.06 | 0.144 | 6.79E-01 |
| ICCBD | Schizophrenia | 0.70 | 0.033 | 1.13E-98 |
| ICCBD | Major depressive disorder | 0.47 | 0.088 | 1.29E-07 |
| ICCBD | Subjective well-being | -0.14 | 0.058 | 1.25E-02 |
| ICCBD | Mean platelet volume | -0.04 | 0.061 | 4.79E-01 |
| PGCBD | Schizophrenia | 0.83 | 0.038 | 5.31E-106 |
| PGCBD | Major depressive disorder | 0.64 | 0.081 | 3.01E-15 |
| PGCBD | Subjective well-being | -0.20 | 0.058 | 4.84E-04 |
| PGCBD | Mean platelet volume | -0.08 | 0.069 | 2.37E-01 |
| ICCBD+PGCBD | Schizophrenia | 0.75 | 0.023 | 6.99E-240 |
| ICCBD+PGCBD | Major depressive disorder | 0.58 | 0.072 | 3.27E-16 |
| ICCBD+PGCBD | Subjective well-being | -0.17 | 0.048 | 3.00E-04 |
| ICCBD+PGCBD | Mean platelet volume | -0.07 | 0.052 | 2.09E-01 |
